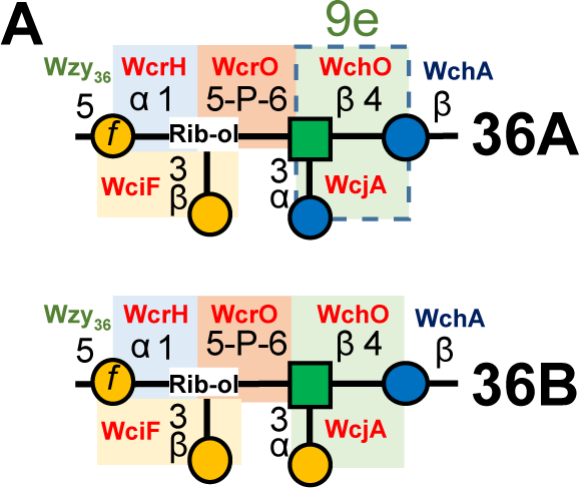# Erratum for Ganaie et al., “Discovery and Characterization of Pneumococcal Serogroup 36 Capsule Subtypes, Serotypes 36A and 36B”

**DOI:** 10.1128/jcm.01756-24

**Published:** 2024-11-27

**Authors:** Feroze A. Ganaie, Jamil S. Saad, Stephanie W. Lo, Lesley McGee, Stephen D. Bentley, Andries J. van Tonder, Paulina Hawkins, Jeremy D. Keenan, Juan J. Calix, Moon H. Nahm

## ERRATUM

Volume 61, no. 4, e00024-23, 2023, https://doi.org/10.1128/jcm.00024-23. Page 6: Figure 2F should appear as shown in this erratum. Residue A in the 36A and 36B capsule polysaccharide repeat units should be “β-D-Glc*p*” instead of “α-D-Glc*p*”.

Page 7: Table 2, rows 1 and 7, “4-α-D-Glc*p*” should read “4-β-D-Glc*p*.”

Page 8: Figure 3A, panels labeled “36A” and “36B” should appear as shown in this erratum.

**Fig 2:**
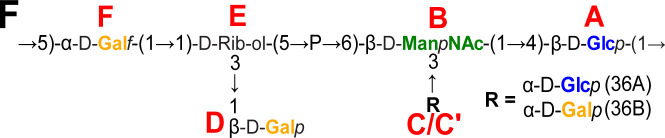


**Fig 3:**